# Associations between shopping patterns, dietary behaviours and geographic information system-assessed store accessibility among small food store customers

**DOI:** 10.1017/S1368980020005017

**Published:** 2022-05

**Authors:** Jared T McGuirt, Qiang Wu, Melissa N Laska, Kimberly P Truesdale, Ann P Rafferty, Ronny A Bell, Alice S Ammerman, Stephanie B Jilcott Pitts

**Affiliations:** 1 Department of Nutrition, University of North Carolina Greensboro, 319 College Avenue, 318 Stone Building, Greensboro, NC 27412, USA; 2 Department of Biostatistics, East Carolina University, Greenville, NC, USA; 3 Division of Epidemiology and Community Health, University of Minnesota, Minneapolis, MN, USA; 4 Department of Nutrition, University of North Carolina at Chapel Hill, Chapel Hill, NC, USA; 5 Department of Public Health, East Carolina University, Greenville, NC, USA

**Keywords:** GIS, Small food stores, Food access, Dietary behaviours, Shopping patterns

## Abstract

**Objective:**

To examine associations between geographic information systems (GIS)-assessed accessibility to small food stores, shopping patterns and dietary behaviours among small food store customers.

**Design:**

Residential addresses and customer shopping patterns (frequency of shopping, and previous purchase of fruits and vegetables) were gathered through customer intercept surveys. Addresses were geocoded, and GIS-assessed distance and driving time from the participants’ residence to the store were calculated. Dietary status and behaviours were assessed using an objective non-invasive measure of skin carotenoids, the National Cancer Institute Fruit and Vegetable Screener, and items to assess sugary beverage intake. Associations between distance and driving time, demographics, shopping frequency, prior reported purchase of fruits and vegetables at the store and dietary behaviours were examined.

**Setting:**

Small food stores (*n* 22) across North Carolina.

**Participants:**

Cross-sectional convenience samples of English-speaking customers aged 18 years or older (*n* 692).

**Results:**

Participants living closer to the small store had lower income and formal education, were more likely to be Black, more likely to have previously bought fruits and vegetables at the store and more frequently shopped at the store. In adjusted models, skin carotenoids (*n* 644) were positively associated with distance to the store from home in miles (*P* = 0·01).

**Conclusions:**

Customers who lived closer to the stores were more frequent shoppers and more likely to have previously purchased fruits and vegetables at the store yet had lower skin carotenoids. These results support continued efforts to examine how to increase the availability and promotion of healthful foods at small food retail stores.

People living in food deserts have reduced access to affordable and nutritious food^(1,2)^. This reduced access can influence shopping habits and dietary patterns and potentially increase the risk of diet-related diseases^(3,4)^. Low-income, rural, and racial and ethnic minorities are often dually burdened with limited access to healthy foods and increased risk of diet-related morbidity and mortality^(1)^. These compounding factors may be contributing to existing diet-related health disparities in these populations^(3)^.

Small food stores, like convenience stores or corner stores, are a routinely accessed component of the retail food environment^(5)^ and have received increased attention as an opportunity to improve access to healthy foods^(6–8)^. These stores often carry less healthy foods and beverages, including processed foods with low nutrient density and added sugars^(9,10)^. The scarcity of healthy foods in small food stores may be exacerbated in rural areas and communities of colour^(10,11)^. Food options are often higher priced, but these stores are also often prevalent in low-income urban and rural locations, and thus more convenient, compared to full-service supermarkets^(12,13)^. For many people living in food deserts, and particularly for those with limited transportation, small food stores may be the sole food venue within a reasonable travel distance^(13)^.

The influence of access to corner stores on shopping and dietary behaviours remains relatively unclear, with intervention studies showing mixed results^(6,8,14)^, and observational epidemiology studies often using incomplete or inadequate measurements (e.g. non-road network Euclidean distance and inappropriate distance measures to account for complex travel patterns)^(3,15,16)^. Relatively few studies have directly examined associations between distance from small food stores to participants’ residential location, purchasing behaviours at specific small food stores, and dietary behaviours^(3,17,18)^. Understanding this relationship is important as efforts are made to increase healthy food options in small food stores.

Prior studies of the association between the food environment and dietary patterns have relied on recall-based dietary assessment measures which are subject to systematic bias and measurement error^(19–21)^. The emergence of a measurement tool to objectively, non-invasively and easily measure skin carotenoid status as an approximation of fruit and vegetable intake has provided a new opportunity to better understand associations between the food environment and dietary behaviours^(22)^. Recent research has examined associations between the food environment and skin carotenoid status, finding that, even when controlling for individual-level variables related to income, age, sex and race/ethnicity, individuals living in areas with higher concentrations of convenience stores (i.e. stores that offer less healthful foods) had lower levels of skin carotenoids^(18)^. To our knowledge, no previous research has examined the associations between objectively assessed proximity to a small food store where customers were shopping, the frequency of shopping, previous purchase of fruits and vegetables at that store, the proportion of foods purchased at the store, and self-reported and objectively measured dietary behaviours. Examining these relationships can help elucidate the influence of accessibility to small food stores located in food deserts on dietary behaviours of customers shopping at the store and can further inform intervention efforts in small food stores. Thus, we sought to examine associations between geographic information systems (GIS)-assessed proximity to stores, customer shopping patterns and dietary behaviours among small food store customers. Distance to the store was hypothesised to be inversely associated with the frequency of shopping such that those who live close to the small food store shop there more frequently and thus purchase a larger proportion of foods/beverages from the small food store.

## Methods

### Study settings and participants

This analysis was conducted as part of a larger study evaluating the impact of the North Carolina Healthy Food Small Retailer Program (HFSRP) on healthy food availability, purchases and consumption^(23)^. The HFRSP was a grant programme to support retailers in stocking nutrient-dense foods (i.e. fresh vegetables and fruits, whole grains, nuts, seeds, beans and legumes, low-fat dairy products, lean meats, and seafood), including funding for refrigeration units and shelving. Data were collected from 22 stores in food deserts (determined using the United States Department of Agriculture (USDA) Food Access Research Atlas dataset^(24)^, with food deserts being defined as low-income census tracts with a substantial number or share of residents with low levels of access to retail outlets selling healthy and affordable foods) across 14 rural- and urban-designated counties in central and eastern North Carolina. Stores were selected as described previously^(15)^.

In brief, stores received HFSRP funding as part of a competitive application process. The study authors did not control the selection of the funded HFSRP stores. Control stores were selected through a matching process so that they would be as similar as possible to HFSRP-funded stores. The study team first matched the stores on a variety of store-level factors including North American Industry Code Standards (NAICS), store type (small grocery or convenience store), store size, and whether the store was located in a census tract that was designated as a USDA food desert (at least 500 persons and/or at least 33 per cent of the population lives more than 1 mile from a supermarket or large grocery store (10 miles, in the case of rural census tracts)^(25)^. For the control stores, the study team then matched on area demographics, including per cent of the census tract that received Supplemental Nutrition Assistance Program benefits and the per cent African American residents in the census tract. All store locations met square footage requirements of the legislation (less than 3000 square feet of heated space) and were authorised retailers in the Special Supplemental Nutrition Program for Women, Infants, and Children (or were willing to undergo authorisation). Some of the stores included a gas pump area, while others functioned as neighbourhood grocery stores. As part of the larger study evaluating the programme, data were collected on the food and beverage items offered at each store as well as customer purchasing and dietary behaviours. Participants were provided a $10 gift card for their time.

Participants were recruited within small stores across 3 years (2017–2019), creating a cross-sectional sample for these analyses. Store customers were approached by research staff after completing their purchase (mostly between 8 am and 5 pm, Monday–Friday). To be eligible for the study, participants had to be 18 years of age or older and speak English. Participants received a study information sheet and provided verbal informed consent before data collection. The *(anonymised)* Institutional Review Board reviewed and approved study #UMCIRB 16-002420. As part of the study, participants completed customer intercept surveys, permitted data collectors to perform bag checks in which the contents of their purchases were recorded and had their skin carotenoids status assessed using a reflection spectroscopy device (the Veggie Meter®, Longevity Link Corp.™)^(26)^.

### Demographic and anthropometric data

Demographic data collected included sex (male, female and other), age (years), race and ethnicity (American Indian or Alaska Native, Asian, Black or African American, Native Hawaiian or Pacific Islander, White, Other or Refused; Hispanic or Latino), highest grade completed in school, annual household income (less than $25 000, $25 000 to $50 000, $50 000 to $75 000, $75 000+ and not reported) and employment status (employed full-time, employed part-time, not employed and retired). We derived BMI (kg/m^2^) from the participant’s self-reported height and weight.

### Customer and store-level geospatial data

As part of the customer intercept survey, participants provided the physical address of their primary residence. Verified small food store addresses were obtained from Reference USA (InfoGroup, 2018; Papillion, NE, USA) business database. All addresses were batch-geocoded to the highest level of accuracy using the Google Maps application programming interface through the BatchGeo^(27)^ website. For additional verification, Google Maps street listings and Google satellite imagery were utilised (Google LLC, Mountain View, CA, USA). Participants with incomplete addresses (no information or street number not provided; the street name was misspelled and could not be determined; and address not found in the geodatabase) were removed from the dataset for this study (*n* 323/1015 (31·8 %)). Those with complete addresses shopped more frequently at the store (*P*<.0001) and had higher sugary beverage consumption (*P* = 0·01) compared to those without a complete address.

The distance (in miles and minutes) from the participants’ residential address to the store where they were surveyed was calculated using the Environmental Systems Research Institute (ESRI) (Redlands, CA, USA) ArcGIS Online network analysis services. This approach uses road networks to simulate typical travel behaviours, particularly by motor vehicle transportation, thus providing a more accurate/realistic estimate of accessibility *v*. distance alone. For accuracy and to reduce edge effects (loss of information due to spatial administrative boundaries like county boundaries), potential participant travel paths along the road network were allowed to traverse administrative boundaries (i.e. county) using a North America-wide road network dataset. Along with physical distance, we generated a time cost variable using ESRI’s predictive traffic modelling to capture the time needed to traverse the shortest road network path based on typical traffic conditions. Census tract-level rural *v*. urban designation for the small food stores and participants were obtained from the USDA Food Access Research Atlas dataset^(24)^ and spatially joined to each store using ArcMap.

### Customer shopping data

Customer shopping data were collected by two mechanisms: customer recall of previous purchases and objective assessment of items purchased at the current visit (bag checks). To determine whether participants had previously purchased fruits and vegetables at the small food store, they were asked the following: ‘Have you ever bought fresh fruits and vegetables from this store?’ To determine the proportion of foods typically purchased at the store, customers were asked ‘Please think about all the foods you eat and all the beverages you drink on a regular basis. About what proportion of all the food and beverages you consume comes from this corner store?’ and were given the response options of ‘All or most of my food and beverages’, ‘More than half’, ‘About half’, ‘Less than half’ and ‘A very small proportion of my food and beverages comes from this corner store’. This question was only asked for 2019 participants.

### Customer dietary data

Customer dietary data were collected using both recall-based and objective measures. The customer intercept survey included the National Cancer Institute (NCI) Fruit and Vegetable (FV) Screener^(28)^, which includes frequency and amount for the following items: 100 % juice (orange, apple, grape or grapefruit); fruit (fresh, canned, frozen and no juice); lettuce salad; French fries/fried potatoes; other white potatoes (baked, boiled, and mashed potatoes, potato salad, and white potatoes that were not fried); cooked dried beans; other vegetables; and tomato sauce (tomato sauce on pasta or macaroni, rice, pizza and other dishes). It also includes frequency for vegetable mixtures (foods such as sandwiches, casseroles, stews, stir-fry, omelets and tacos). We used the NCI’s standard scoring algorithms to calculate FV servings per day^(28)^.

To determine sugar-sweetened beverage (SSB) intake, we used items adapted from the Behavioral Risk Factor Surveillance System survey^(29)^. Participants were asked about the frequency of ‘regular soda’ consumption (not including diet soda or diet pop) and sweetened fruit drink consumption (including Kool-Aid®, cranberry and lemonade). Response options included never; 1–3 times/month; 1–2 times/week; 3–4 times/week; 5–6 times/week; 1 time/d; 2 times/d; 3 times/d; 4 times/d and more than 5 times/d.

Skin carotenoid levels were collected from participants using a reflection spectroscopy device called the ‘Veggie Meter®’, a valid, non-invasive, objective approximation of fruit and vegetable intake^(30)^. Participants provided finger scans three times (which were averaged) to generate skin carotenoid scores, which range from 0 to 800, with higher scores indicating higher skin carotenoid levels and thus greater FV intake. This tool has been validated against plasma carotenoids in a sample of Black and White participants in eastern North Carolina (correlation between plasma and Veggie Meter ® assessed skin carotenoids was 0·71, *P* < 0·0001)^(30,31)^. The mean in our prior study was 234·2 (sd = 86·2)^(32)^.

### Analysis

Univariate statistics, including means and frequencies, were generated for demographic variables, GIS distance variables, customer shopping patterns and customer dietary behaviours. The GIS-derived distance distribution was right-skewed, so a base 2 log transformation of travel distance and time was generated. For bivariate analysis, we examined correlations between travel distance and travel time with skin carotenoids, and recall-based fruit, vegetable and SSB consumption.

**Fig. 1:**
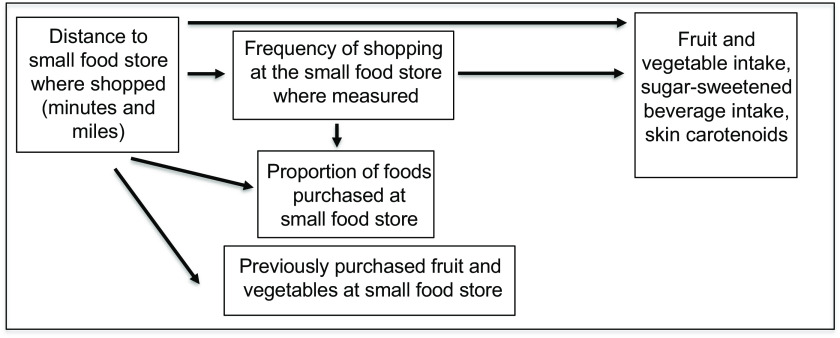
Tested hypothesised associations between distance, shopping behaviours and dietary intake

Figure 1 demonstrates the hypothesised associations between distance, shopping behaviours and dietary intake. Distance to the store was hypothesised to be inversely associated with the frequency of shopping such that those who lived close to the small food store shop there more frequently and thus purchased a larger proportion of foods/beverages from the small food store. Specifically, as noted in Fig. 1, we examined (1) if the frequency of shopping at the small food store, the proportion of all their food purchases coming from the small food store relative to other stores, and previously purchased fruit and vegetables at the small food store were associated with travel distance from the residential address to the small food store (miles and minutes) and (2) if travel distance from the residential address to the small food store (miles and minutes) and frequency of shopping at the small food store were associated with skin carotenoids and self-reported diet (FV and SSB intake).

We used a general logistic regression with random effects to examine the association between shopping frequency using covariates (year, age, sex, race/ethnicity, urban/rural and employment), and travel distance. The small food store was included as a random effect to adjust for potential clustering. Using a linear mixed model, we examined (1) if travel distance (miles and minutes) was associated with skin carotenoids and self-reported diet (FV and SSB intake) and (2) if shopping frequency was associated with skin carotenoids and self-reported diet (FV and SSB intake). Covariates for the full regression models included age (in years), sex (male or female), survey year, race/ethnicity (non-Hispanic White or other race/ethnicity), urban *v*. rural residence and employment (Yes/No). For missing covariate data, pairwise deletion (available-case analysis) was used to minimise data loss, increase power and avoid potential bias in parameters and estimates that can result from listwise deletion methods^(33)^. Additionally, we conducted a mediation analysis to examine the frequency of shopping as a mediator between distance and dietary outcomes while controlling for covariates using SAS Proc Causalmed. We treated shopping frequency as a continuous mediator (SAS Causalmed procedure does not allow categorical mediators with more than two levels). We also tested interactions of urbanicity with shopping frequency and distance to store in all models but found that none were statistically significant, so they were removed from the models. Sensitivity analyses were completed for participants residing within 10 miles (*n* 523) and 50 miles (*n* 677) of the store for all models, but findings were consistent with models including all participants, so we report results of models containing all participants. All analyses were conducted using SAS version 9.4 (SAS Institutes).

## Results

The characteristics of the study participants are found in Table 1. There were 692 participants with complete address data (372 participants in 2017, 160 in 2018 and 160 in 2019) across the 22 stores. The average participant age was 43·5 years (sd = 15·2). Most participants were male (56·7 %) and/or Black (64·6 %). Black males made up the highest percentage of the sample (37·2 %) for combined race/ethnicity and sex. Nearly, half of participants (40·5 %) had an annual household income of less than $25 000, most were high school graduates or less (62·4 %) and 40·6 % were unemployed. The average BMI was 29·5 (sd = 7·4) kg/m^2^.

**Table 1:** Summary of sociodemographic and dietary characteristics of study participants

Characteristics	*n*	%	Mean	sd
Age (years)	690		43·5	15·2
BMI (kg/m^2^)	678		29·5	7·4
Skin carotenoids	672		235·8	84·3
National Cancer Institute-Screener-assessed fruit and vegetables (servings/d)	678		3·8	3·4
Sugar-sweetened beverages (times/d)	692		2·1	2·4
Distance to small food store (miles)	692		8·5	14·0
Distance to small food store (min)	692		13·7	18·3
Sex				
Female	296	42·8		
Male	392	56·7		
Missing	4	0·6		
Race				
Black	447	64·6		
White	171	24·7		
Other	71	10·3		
Missing	3	0·4		
Race and sex				
Black/male	255	37·2		
Black/female	191	27·8		
White/male	100	14·6		
White female	70	10·2		
Other/male	36	5·3		
Other/female	34	4·9		
Income				
<$25 000	280	40·5		
≥$25 000	328	47·4		
Not reported	84	12·1		
Education level				
At least some college	258	37·3		
High school graduate or less	432	62·4		
Missing	2	0·3		
Employment status				
Currently employed	406	58·7		
Not working	281	40·6		
Missing	5	0·7		
Distance from home to small food store				
Live within 1 mile of store	251	36·2		
Live >1 mile from store	441	63·8		
Ever bought fruit and/or vegetables from small food store (*n* 492)				
Yes	182	37·0		
No	294	59·8		
Missing	16	3·2		
Shopping frequency at small food store where surveyed				
1–2x/week or less	227	32·8		
3–6x/week	166	24·0		
1x/d or more	283	40·9		
Missing	16	2·3		
Self-reported proportion of foods purchased at the small food store (2019 sample only, *n* 146)				
All or most of my foods	17	11·7		
More than half	16	11·0		
About half	31	21·2		
Less than half	31	21·2		
A very small proportion	51	34·9		

### Customer shopping

Most participants (59·8 %) had not purchased a fresh fruit or vegetable previously from the small food store where they were surveyed (previous visit). Most of the participants surveyed were frequent shoppers of the store, with 40·9 % shopping one or more times per day. Of the year 2019 participants who answered the question regarding the proportion of foods purchased at the store, 34·9 % answered that a ‘very small proportion of my food and beverages comes from this corner store’, with 11·7 % responding that ‘All or most of my food and beverages’ were purchased at that small food store. Almost half of the year 2019 participants (43·9 %) stated that half or more of their food and beverages come from the store where surveyed.

### Customer diet

Participants reported consuming an average of 3·8 (sd = 3·4) servings of fruits and vegetables per day (based on the NCI FV screener) and consuming SSB 2·1 (sd = 2·4) times per day. The average Veggie Meter® reading was 235·8 (sd = 84·3).

### Distance to the store

The average distance to the store where surveyed from the participant’s home was 8·5 (sd = 14·0) miles. A little over one-third (36·2 %) of participants lived within one mile of the store, and about one-quarter (24·4 %) of participants lived greater than 10 miles from the store. The range was from 0·01 to 98·9 miles. The average travel time to the store where participants were surveyed from their home was 13·7 min (sd = 18·2), with an average travel time of 9·3 min (sd = 14·3) for urban participants, and 21·7 min (sd = 21·6) for rural participants.

### Distance to the store and demographic variables

A summary of the relationship between distance to the store and demographic variables is found in Table 2. In unadjusted bivariate analyses, Black participants lived significantly closer to the store than White or ‘other’ racial/ethnic groups in both miles (*P*<.0001) and minutes (*P*<.0001). Those making less than $25 000/year lived significantly closer to the store than those making ≥$25 000/year (*P*<.0001 for miles and minutes). Participants who were not employed lived significantly closer to the store than employed participants (*P*<.0001 for miles and minutes).

**Table 2:** Associations between distance and customer sociodemographic variables, store-level variables and shopping behaviours among customers

	Miles		
	Distance	sd	*P*
Sociodemographic variables			
Race (*n* 689)			
Black	6·2	11·8	<·0001
White	14·6	17·4	
Other	8·5	13·7	
Income (*n* 692)			
<$25 000	6·1	12·1	<·0001
$25 000+	11·2	15·5	
Not reported	5·8	11·8	
Employment (*n* 687)			
Not employed	5·8	11·8	<·0001
Employed	10·4	15·1	
Store-level variables			
Urbanicity (*n* 692)			
Urban	6·0	12·9	<·0001
Rural	14·4	14·6	
Shopping behaviours			
Previously bought fruit or vegetables at participating store (*n* 476)			
Yes	4·6	8·6	<·0001
No	10·0	15·3	
Shopping frequency (*n* 676)			<·0001
1–2x week or less	13·1	16·1	
3–6x week	7·2	12·8	
1x/d or more	4·9	10·7	

### Distance to the store and store-level variables

There was a statistically significant difference in distance between the residential address and the store in miles (*P*<.0001) and minutes (*P*<.0001) for participants shopping at rural *v*. urban stores, with rural stores having customers who lived further from the store where participants were surveyed (Table 2). The mean distance to the store for urban participants was 6·0 miles (sd = 12·9), and for rural participants, the mean distance to the store was 14·4 miles (sd = 14·6).

### Associations between distance to the store and shopping patterns

A summary of the results of the associations between distance to the store and shopping patterns is included in Table 2. Participants that had previously bought FV at the store lived a shorter distance in miles (*P*<.0001) and minutes (*P*<.0001) from the store than those who had not previously bought FV at the store. There was a statistically significant association between shopping frequency and the distance to the store in miles (*P* = <.0001) and minutes (*P* = <.0001), indicating that participants who lived closer to the store more frequently shopped at the store.

### Associations between distance to the store and proportion of foods purchased

Those with a higher proportion of all their food purchases coming from the small food store relative to other stores lived closer to the store (2019 sample only, *n* 146), though this relationship was not statistically significant ((*F*(4, 141) = 2·10, *P* = 0·08), Table 3).

**Table 3:** Summary of measures of distance to the small food store where shopping from home (miles, minutes) and proportion of foods and beverages purchased among *n* 146 customers self-reporting in 2019

Proportion of foods and beverages purchased	*n*	Miles*	
		Mean	sd
All or most of my food and beverages	17	2·4	2·4
More than half	16	7·6	12·9
About half	31	4·3	6·9
Less than half	31	6·0	8·8
A very small proportion	51	10·5	18·3

* *F* (4, 141) = 2·10, *P* = 0·08.

### Distance to the store and frequency of shopping

In a general logistic regression model controlling for year, store, age, sex, race/ethnicity, employment and rural *v*. urban residence, frequency of shopping at the small food store where surveyed was inversely associated with distance in miles to the store where surveyed such that individuals (*n* 664) who lived further from the store shopped less frequently (estimate = 0·22, se = 0·058, *P* =< 0·001 for 3–6x/week *v*. 1–2x/week or less) (estimate = −0·16, se = 0·049, *P* = 0·001 for 1x/d or more *v*. 3–6x/week) (Table 4).

**Table 4:** Associations between frequency of shopping at the small food store where surveyed, and distance to the store in miles with objectively measured skin carotenoids, self-reported fruit and vegetable intake, and self-reported sugar-sweetened beverage intake*

Outcome		Independent variable					
		Distance to store			Frequency of shopping		
		Parameter estimate	se	*P* value	Parameter estimate	se	*P*
Frequency of shopping at the store (*n* 664)	1–2x/week or less *v*. 3–6/week	0·22	0·058	<·001			
	1x/d or more *v*. 3–6/week	−0·16	0·049	0·001			
Skin carotenoids (*n* 644): direct effect/mediated effect		4·12†/3·86‡	1·55/1·61	0·01/0·02	20·4 (1–2x/week or less *v*. 3–6/week)	8·7	0·02
					14·5 (1x/d or more *v*. 3–6/week)	8·3	0·08
Fruit and vegetable intake (NCI Screener) (*n* 651): direct effect/mediated effect		−0·066†/-0·053‡	0·055/0·057	0·23/0·35	−0·42 (1–2x/week or less *v*. 3–6/week)	0·36	0·25
					−0·12 (1x/d or more *v*. 3–6/week)	0·35	0·72
Sugar-sweetened beverage intake (*n* 664): direct effect/mediated effect		0·033†/0·058‡	0·041/0·041	0·41/0·17	−0·41 (1–2x/week or less *v*. 3–6/week)	0·25	0·09
					0·21 (1x/d or more *v*. 3–6/week)	0·24	0·37

NCI Screener, National Cancer Institute Screener.

* Controlling for year, age, sex, race, income, urbanicity (urban *v*. rural) and employment, with store included as a random effect.

† Total effect.

‡ Direct effect.

### Frequency of shopping and dietary measures

In a linear mixed model controlling for year, store, age, sex, race/ethnicity, employment status, rural *v*. urban residence and distance to store, participants (*n* 644) who shopped 1–2 per week or less had significantly higher skin carotenoids compared to those who shopped 3–6 times per week (estimate = 20·4, se = 8·7, *P* = 0·02) (Table 4). There were no statistically significant differences found across levels of shopping frequency with self-reported NCI FV intake (*n* 651) or SSB intake (*n* 664). (Table 4)

### Distance to the store and dietary measures

In the linear mixed model with skin carotenoids as the dependent variable of interest (*n* 644), controlling for year, store, age, sex, race/ethnicity, employment status, rural *v*. urban residence and shopping frequency, skin carotenoids were positively associated with distance to the store from home in miles (estimate = 3·86, se = 1·61, *P* = 0·02) (Table 4). There were no statistically significant associations between self-reported FV intake (*n* 651) and SSB intake (*n* 644) and distance to the store (*P* = 0·35 and *P* = 0·17, respectively).

### Shopping frequency as a mediator between distance and dietary measures

The percentage mediated effect of distance to the store was 6·0 % (*P* = 0·55) on skin carotenoids, 19·9 % (*P* = 0·49) on NCI FV intake and −109·0 % (*P* = 0·43) on sugary beverage intake, indicating that shopping frequency was not found to be a statistically significant mediator between distance and dietary measures. Thus, our original hypothesis of shopping frequency being a mediator between distance and dietary measures was not supported.

## Discussion

Customers in our study who lived closer to the small food stores had lower incomes, were more likely to be unemployed, shopped more frequently at the store and had lower skin carotenoids (indicative of lower carotenoid fruit and vegetable consumption), yet they were more likely to have previously purchased FV at the store where surveyed. This could be due to increased exposure from more frequent shopping at the store and possibly relying on the store for most or all of their household food and beverage needs. The mean carotenoids score (235·8 (sd = 84·3)) was similar to that in our prior study of an earlier time point (234·2 (sd = 86·2)). Our findings are also aligned with the findings of others regarding mean Veggie Meter® scores of about 200–250^(34–37)^. The Veggie Meter score is suggestive of about 4 weeks of exposure^(38)^. The fact that carotenoid scores were lower for those that lived closer to the small store, yet they were more likely to have previously purchased FV at the store where surveyed, may suggest that without access to FV at small stores, skin carotenoid levels could be even lower among this population. The lower carotenoid scores among those who live closer and shop more frequently at the small store where surveyed could indicate that these residents are getting most, or all of their household food needs met at small stores. This could also suggest they are more dependent on the store for their basic shopping due to a limited income and the need to shop more proximally to their residence rather than at stores with a wider selection. With limited food availability at the small store, they were less likely to get fruits and vegetables and thus had lower carotenoid scores. This supports efforts to make the food environment in such stores as healthy as possible. In a previous study of skin carotenoid status and distance to and frequency of shopping among supermarket customers, we found no statistically significant difference in skin carotenoids by frequency of shopping or by distance to the store, though we did find that proximity to less healthy stores such as convenience stores and small grocery stores was associated with lower skin carotenoid levels^(18)^. These findings suggest that public health efforts should continue to promote healthier foods and beverages in similar small food stores.

We found that people living closer to the store where they were surveyed tended to shop there more often and purchased the highest proportion of foods from that store when compared to customers living further from the store. This idea is consistent with the distance decay theory, which suggests that the interaction among two entities decreases as distance increases^(39)^. Our finding that shopping frequency was not a statistically significant mediator between distance to the store and dietary outcomes, but that frequency was statistically significantly associated with distance, may suggest that the influence of distance and frequency are interconnected but also distinctly relevant. Future research should examine this more closely. While previous research has suggested that the closest food retail venue (particularly for supermarkets) is not necessarily where customers most frequently shop^(40)^, partially due to the complexity of food shopping decisions^(41)^, it does appear that proximity to small food stores, in particular, could be an influential factor in usage^(18)^. We found that those making less than $25 000/year and those who were unemployed lived closer to the small food store where surveyed, which suggests that these types of stores are readily accessible to people with lower socio-economic status, who may also have disproportionately worse health outcomes compared to those of higher socioeconomic status^(42)^.

In addition to proximity being associated with shopping frequency, our results suggest that Black male participants (37·2 % of our sample) were frequent shoppers at the small food stores. This is a priority population since they bear a disproportionate burden of chronic disease^(43)^ and pre-mature mortality^(43)^ in the USA but have received relatively little attention in the public health and food access literature. Future research should try and better understand the influence of food access, particularly in small food stores, on Black male shopping and dietary behaviours.

The strengths of the study include the large sample size and the use of both objective and recall-based dietary measurements, the use of GIS to derive multiple objective measures of proximity based on road network data and the examination of shopping behaviours and preferences in combination with GIS-derived objective access measures. Despite its strengths, this study has limitations that should be acknowledged when interpreting these results. We used a convenience sample and a cross-sectional study design, which may limit generalisability and limit the ability to determine causation. There may be selection bias based on the customers and stores who chose to participate (those more likely to participate may also be more interested in health or the participant incentive) and those with complete address information. We found that those with complete addresses shopped more frequently at the store and had higher sugary beverage consumption, so our results for these variables could be biased away from the null. In a few cases, customers completed surveys without purchasing a food or beverage item. Also, participants were encouraged but not required to wash their hands before skin carotenoids were assessed using the Veggie Meter®, which could lead to less accurate readings^(43)^. We did not assess physical access from other important locations of daily life (like work, childcare and other shopping), nor do we know the mode of transportation used for the customer’s visit to the store. We also did not have information about shopping patterns at other food venues.

## Conclusions

This study contributes to our understanding of food accessibility, shopping patterns and dietary outcomes among small food store customers using objectively measured distance and dietary outcomes. We found that living closer to the small food store where individuals shop is associated with more frequent shopping and may also be associated with lower dietary quality. This provides evidence of the potential influence of small food stores on dietary behaviours, particularly for those populations who are more reliant on this store type for their food and beverage purchases. From an intervention and policy perspective, our findings support attempts to promote healthier purchases in small food stores by increasing the availability of healthier food options, given that individuals of lower socio-economic status live closer to and shop more frequently at these stores. Future interventions and healthy food store programme and policy planning should take into account these complex relationships to optimise public health impact.
